# Supplementary material for: Biocompatibility Analysis of Bio-Based and Synthetic Silica Nanoparticles during Early Zebrafish Development
Source: Int J Mol Sci. 2024 May 18;25(10):5530. doi: 10.3390/ijms25105530 (PMC11121961; doi:10.3390/ijms25105530)
Supplement: Supplementary file 1 [file ijms-25-05530-s001.zip › ijms-2986500-supplementary.pdf]

Table S1. Primer sequences of genes evaluated in this paper

Neurotoxicity	Forward (5′-3′)	Reverse (5′-3′)
rpl13a	TCTGGAGGACTGTAAGAGGTATGC	AGACGCACAATCTTGAGAGCAG
β-synuclein	ATGGATGTTTTTATGAAGGGGC	ACGCTGTCTTTGGTCTTGCT
parkin	GCGAGTGTGTCTGAGCTGAA	ATCACAGCCCTGAAGTGTGG
pink1	GGCAATGAAGATGATGTGGAAC	ATCACGTTGGGATGAGCACT
uch-l1	CTTCCCTTTAACGCAACAGC	CCCACAGTTCCACAGGAGTT
park7	TGTTACTGTCGCAGGTCTGG	CAGGCAGAAGAACAACGTCA
atg5	AGGGGATAACAGCACAAACG	CTTCTTATGCAGCGTGTCCA
ambra1a	TAACCAGGAAACTGGCCAAC	AATATGCTGCAGGGGACAAC
gfap	GGATGCAGCCAATCGTAAT	TTCCAGGTCACAGGTCAG
gap43	CAGCCGACGTGCCTGAA	GGATTCCTCAGCAGCGTCTG
**Inflammation and oxidative stress**		
Il8	GTCGCTGCATTGAAACAGAA	CTTAACCCATGGAGCAGAGG
cxcl-cic	CATCCGGCCAGCTCTGCTTGAAT	CCACTCTTGACCTCCTGTGCTCTCT
ccl34a.4	CTTTGACGCATGGAGGATTT	TGCAGCTCAACCAGAAGATG
nfkb2	TGGCTGGAGCACTAAGGATG	CCTCTCTGCTTTGGCTCCTC
nfkbiaa	ACAACCGAAGAGAGAACATGGA	CGAAATCTCCCGCGTCTCAT
tnfα	CGCTGGTGATGGTGTCTAGG	CCCTGGGTCTTATGGAGCGT
il1β	CGTACTCAAGGAGATCAGCGG	GCGGTGCTGATAAACCAACC
il6	GGCATTTGAAGGGGTCAGGA	GCGTTAGACATCTTTCCGTGC
hmox1a	CCACGTCAGAGCTGAAAACA	AGCGCTCGGTAGATCTCGTA
nrf2a	GAGCGGGAGAAATCACACAGAATG	CAGGAGCTGCATGCACTCATCG
nrf2b	GGCAGAGGGAGGAGGAGACCAT	AAACAGCAGGGCAGACAACAAGG
**Housekeeping**		
ism12b	AGTTGTCCCAAGCCTATGCAATCAG	CCACTCAGGAGGATAAAGACGAGTC

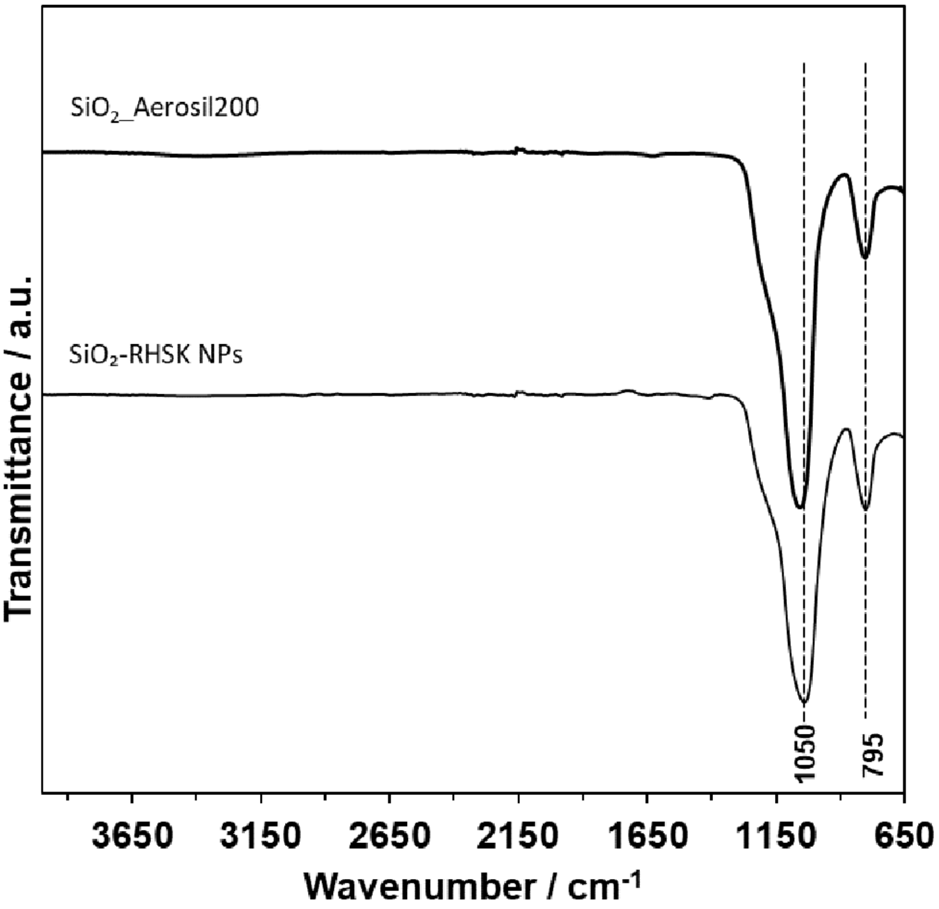

Figure S1. FTIR-ATR spectrometry. The FTIR spectra for both samples exhibited bands at ca. 800 and 1050 cm^{-1}, which can be assigned to the symmetric and asymmetric stretching vibration of Si-O-Si bonds, respectively. As expected, similar spectra were obtained for SiO_2-RHSK NPs and SiO_2-Aerosil200.

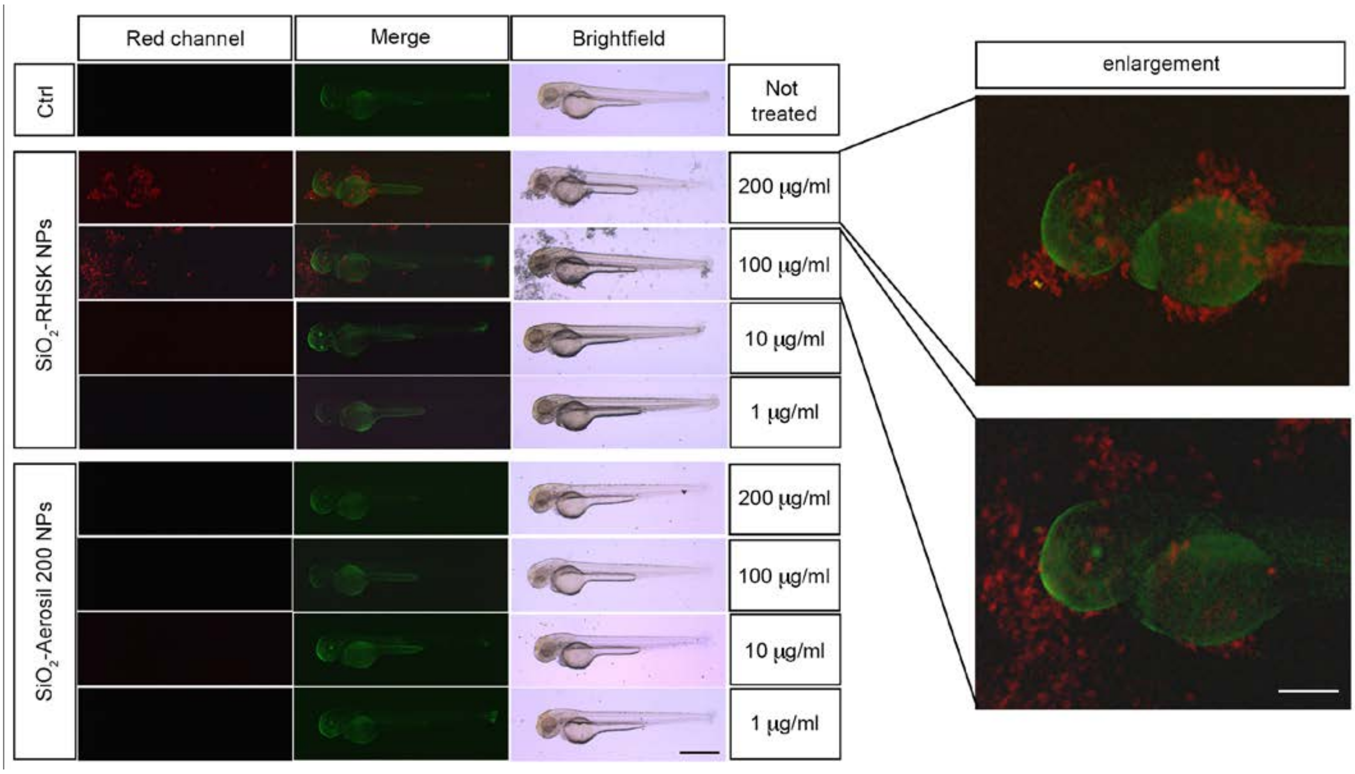

Figure S2. Representative images of embryos at 72 hpf exposed to SiO_2-RHSK and SiO_2-Aerosil200 NPs at different concentrations for 48 h, showing visible SiO_2-RHSK NPs agglomerates under a fluorescent stereomicroscope.
To evaluate the NPs behavior after suspension in the FET solution, embryos exposed to SiO_2-Aerosil200 and SiO_2-RHSK NPs at different concentrations were observed under a fluorescent stereomicroscope. The magnifications showed that agglomerated of SiO_2-RHSK NPs are visible around the embryos at 72 hpf developmental stage, especially at higher concentrations (100 and 200 µg/ml). Scale bar: 100 µm.
